# Concentrations of cardiac Troponin I before and after ovariohysterectomy in 46 female dogs with pyometra

**DOI:** 10.1186/1751-0147-50-35

**Published:** 2008-09-11

**Authors:** Lena Pelander, Ragnvi Hagman, Jens Häggström

**Affiliations:** 1 University Teaching Hospital, Swedish University of Agricultural Science, Uppsala, Sweden; 2 Department of Clinical Sciences, Swedish University of Agricultural Science, Uppsala, Sweden

## Abstract

**Background:**

Canine pyometra is a common disease in countries where routine spaying of young dogs is not common practice. This disease is known to lead to systemic inflammation potentially affecting multiple organs in the body, including the heart. Cardiac-specific Troponin I (cTnI) is a sensitive marker of myocardial cell damage, which can result from ischemia, trauma, toxins or inflammation. Dogs with pyometra are also exposed to anaesthesia which can potentially result in myocardial cell damage. The aims of the study were 1) to evaluate the occurrence of myocardial cell damage as indicated by increased serum concentrations of cTnI in dogs with pyometra and relate these to presence of systemic inflammation and 2) to evaluate the change in cTnI-concentrations after anaesthesia and surgery.

**Methods:**

Serum cTnI concentration was measured preoperatively and one day after surgery in 46 female dogs with pyometra and 15 female dogs that underwent surgery for other reasons (ovariohysterectomy and mammary tumours).

**Results:**

Forty-six female dogs of different breeds diagnosed with pyometra were included. The dogs had a median age of 8.5 years (IQR 7.5–10) and a median weight of 29 kg (IQR 9–32). Of the 46 dogs, 37 (80%) fulfilled the chosen criteria for systemic inflammatory response syndrome (SIRS) at inclusion. Thirteen (28%) of the dogs had increased cTnI concentrations (> 0.2 μg/l) before surgery and 18 (39%) had increased cTnI-concentrations the day after surgery. The cTnI concentrations in the 13 dogs with increased preoperative cTnI concentrations decreased in 8 dogs, increased in 4 dogs, and was unchanged in one dog. Seven dogs with nondetectable preoperative cTnI concentrations had increased postoperative concentrations. The only significant association between the studied laboratory or clinical variables (including SIRS) and cTnI concentration was preoperative percentage band neutrophils (PBN) and postoperative cTnI concentration (P = 0.016). In total, 20 dogs (43%) had increased pre- or postoperative cTnI concentrations. Seven dogs (15%) had pre-or postoperative concentrations of cTnI of 1.0 μg/l or higher.

**Conclusion:**

Mild to moderate increases in cTnI appears to be common in dogs with pyometra before and after surgery, but the clinical importance of this finding is uncertain. None of the studied clinical variables were found to reliably predict increased preoperative cTnI concentrations. Because of the pre- and postoperative variation in cTnI concentrations, it was not possible to identify a negative effect of anaesthesia and surgery on myocardial cell integrity.

## Background

Myocardial damage can be caused by multiple conditions including ischemia, trauma, toxins or inflammation. Cardiac-specific Troponin I (cTnI) is currently the most sensitive and specific marker of myocardial cell damage in the dog [1,2]. Cardiac-specific Troponin I is a protein that is expressed at high concentrations only in the myocardium. When cardiac myocytes are damaged, cTnI leaks into the bloodstream and can be detected in serum [1,3]. In normal dogs serum concentrations of cTnI are low or, most often, undetectable [4].

Canine pyometra is a common disease in countries where routine spaying of young dogs is not common practice. The condition may cause systemic inflammation, which may potentially damage multiple organs in the body, including the heart [5]. The presence of systemic inflammatory response syndrome (SIRS) may be predicted by certain clinical and laboratory parameters [6]. The systemic inflammation can, if not successfully treated, progress to multiple organ dysfunction syndrome (MODS) and death [7].

The safest and most effective treatment for canine pyometra is ovariohysterectomy [8]. However, anaesthesia and surgery may cause myocardial ischemia with subsequent myocardial cell damage, especially in individuals with systemic inflammation and impaired circulation [9]. In humans, the occurrence of perioperative ECG abnormalities and "silent myocardial ischemia" is well recognised [10]. One study documented myocardial ischemia 12 hours postoperatively (as measured by increased concentrations of cTnI) in healthy women undergoing caesarean section [11]. It has been demonstrated that perioperative elevations of cTnI concentrations were associated with major cardiac complications up to 1 year after surgery [12].

Thus, myocardial injury is a potential cause of increased morbidity and mortality in dogs with pyometra before, during and after surgery. However, there are no reports concerning the occurrence and significance of perioperative myocardial damage in dogs undergoing anaesthesia irrespective of the underlying condition. The presence of myocardial damage may often be overlooked and difficult to detect when suspected if it does not lead to compromised cardiac function, arrhythmias or regional abnormal ventricular motion. Analysis of the serum concentration of cTnI can reveal both clinical and subclinical damage to the myocytes [9,13].

The aims of the present study were 1) to evaluate the occurrence of myocardial cell damage as indicated by increased serum concentrations of cTnI in dogs with pyometra and relate these to the severity of systemic inflammation and other clinical variables and 2) to evaluate the change in cTnI concentrations after anaesthesia and surgery.

## Materials and methods

This study was approved by the Uppsala County local ethical committee.

### Dogs

Forty-six female dogs diagnosed with pyometra were recruited to the study at the Department of Small Animal Clinical Sciences, Swedish University of Agricultural Sciences (SLU), Uppsala between January 2004 and December 2005. At the time of arrival, a physical examination was performed on dogs presenting with a history compatible with pyometra (polyuria, polydipsia, anorexia, vomiting, lethargy, fever, vulvar discharge, recent oestrus). Blood samples analysed for complete blood count (CBC) and cardiac-specific Troponin I (cTnI) were collected and radiography and/or ultrasonography of the abdomen was performed, and dogs diagnosed with pyometra were included in the study. Dogs whose owners did not agree to ovariohysterectomy, and dogs with clinical or laboratory findings indicative of other organ-related or systemic disease were excluded from the study. Recorded data obtained from the case history and physical examination included age, weight, rectal temperature, heart rate and respiratory rate. A second serum sample for analysis of cTnI was collected 12–24 hours after surgery.

Fifteen female dogs undergoing surgery for neutering (n = 12) or tumour mammae (n = 3) were recruited as control dogs. None of these dogs had a history or clinical signs indicative of other disease, and in the case of tumour mammae thoracic radiographs had shown that the dogs were free of visible pulmonary metastases. A physical examination was performed on all of these control dogs. Serum for analysis of cTnI was collected at presentation and 12–24 hours after surgery.

### Diagnosis of pyometra

Abdominal radiography and/or ultrasonography were performed on all dogs. The radiological examination included left and ventrodorsal projections of the entire abdomen using the standard procedure at the Section for Diagnostic Imaging, Department of Clinical Sciences, SLU, Sweden. The diagnosis of pyometra was established when an enlarged (and, if ultrasonography was performed, fluid-filled) uterus was found, as previously described [14,15]. The diagnosis was confirmed by the presence of an enlarged uterus containing pus during the surgical procedure.

### Diagnosis of inflammatory response syndrome (SIRS)

Dogs were grouped into two groups; SIRS-positive and SIRS-negative. Dogs that fulfilled two or more of the following criteria were considered SIRS-positive: 1) Resting heart rate > 120/min; 2) respiratory rate > 20/min; 3) rectal temperature above 39.2°C or below 38.1°C; and 4) total white blood cell count (WBC) above 16 × 10^9 ^or below 6 × 10^9 ^cells per l blood or more than 3% band neutrophils [6].

### Sample handling

Blood samples for hematological and biochemical analysis were taken from the distal cephalic vein into EDTA and serum Vacutainer^® ^tubes (Becton & Dickinson, Meylon Cedex, France) and transported to the laboratory for analysis within one hour of collection.

### Ovariohysterectomy

The dogs were premedicated with glycopyrrulate, methadone, acepromazine and carprofen. Anaesthesia was induced with propofol and maintained with isoflurane. In dogs that were considered an anaesthetic risk because of a severely compromised general condition (n = 5) anaesthesia was induced with diazepam and ketaminol and maintained with isoflurane. Ovariohysterectomy was performed using a standard ventral midline approach [16]. The procedure was performed within 24 hours in all dogs except one. The owners wished to delay surgery until after the weekend in this dog because of a good general condition. All dogs were treated with iv fluids before, during and after surgery. Approximately half of the dogs were treated with antibiotics perioperatively. All dogs received preoperative and postoperative opioids until discharge. No medications were given before the preoperative blood samples were collected.

### Haematology, cTnI analysis and blood biochemistry

The CBC was performed using Abbott CELL-DYN 3500 (Abbott Diagnostics, Illinois, USA) in combination with manual microscopy in all cases except eight, where the haematology was performed using the QBC Vet Autoread (IDEXX Laboratories, Maine, USA). In these eight cases the CBC was performed in an emergency situation when the QBC Vet Autoread was the only option for analysis of haematology.

Troponin I was analyzed using a commercially available method (IMMULITE Troponin I, Diagnostic Products Corporation, Los Angeles, USA). This is an immunometric method where antibodies raised against human cTnI bind to existing cTnI in the sample. The lower limit of detection for the cTnI assay is 0,2 μg/l. The possibility of using this method for detection of cTnI in serum samples from dogs was investigated in an earlier study [17,18]. The upper reference limit for normal dogs in our laboratory was 0.2 μg/l.

Adjunct serum biochemical analyses were not included in the study protocol but were performed in some cases, preoperatively (Table 1). ALT, ALP, creatinine and glucose were analyzed using a commercially available method (IDEXX VET TEST Chemistry Analyzer, IDEXX Laboratories, Maine, USA).

**Table 1:** Median and interquartile ranges (IQR) of preoperative plasma biochemical variables (ALT, ALP, creatinine and glucose concentrations) in the study population of dogs with pyometra.

**Biochemical variable**	**Median**	**IQR**	**Reference interval**
**ALT **(u/l) (n = 27)	16	10–36	10–100
**ALP **(u/l) (n = 23)	162	87–229	23–212
**Creatinine **(μmol/l) (n = 33)	82	67–100	44–159
**Glucose **(mmol/l) (n = 20)	6.8	5.9–7.1	4.3–6.9

### Statistics

All statistical analyses were performed using a statistical programme (JMP v 5.0, SAS, Cary, USA). Serum concentrations of cTnI from SIRS-positive and SIRS-negative dogs were compared using Wilcoxon Rank Sum Test.

The association between cTnI concentrations and haematological and blood-biochemical variables, and variables obtained from the physical examination were evaluated by a Spearman rank correlation. Values are reported as the median and interquartile range (IQR). The significance level was set at p < 0.05.

## Results

### Dogs with pyometra

The median age of the 46 dogs with pyometra at presentation was 8.5 years (IQR 7.5–10) and median body weight 29 kg (IQR 9–32). The group comprised 8 mongrel dogs and 38 dogs of 22 different breeds.

### Control dogs

The median age of the dogs in the control group was 5 years (IQR 2–8). Median body weight was 27 kg (IQR 20–32). The group comprised 3 mongrel dogs and 12 dogs of 12 different breeds.

### Preoperative cTnI-concentrations

All of the dogs in the control group had undetectable preoperative cTnI concentrations (Table 2). Of the 46 dogs with pyometra, 13 (28%) had increased preoperative cTnI concentrations (range 0.3–13.2 μg/l) (Table 3).

**Table 2:** Age, weight, reason for surgery (n = neutering, tm = tumor mammae) and pre-and post-operative serum cTnI-concentrations in 15 female dogs undergoing elective surgery (control group).

Case No	Age (years)	Weight (kg)	Reason for surgery	Pre-operative cTnI (μg/l)	Post-operative cTnI (μg/l)
1	9	28	n	< 0.2	< 0.2
2	4	38	n	< 0.2	< 0.2
3	6	22	n	< 0.2	< 0.2
4	4	8	n	< 0.2	0.4
5	5	20	n	< 0.2	< 0.2
6	9	31	tm	< 0.2	< 0.2
7	8	18	tm	< 0.2	< 0.2
8	7	22	n	< 0.2	< 0.2
9	9	10	tm	< 0.2	< 0.2
10	4	35	n	< 0.2	3.0
11	5	32	n	< 0.2	< 0.2
12	1	57	n	< 0.2	< 0.2
13	1	26	n	< 0.2	< 0.2
14	1	27	n	< 0.2	< 0.2
15	2	30	n	< 0.2	< 0.2

**Table 3:** Weight, age, white blood cell count (WBC), percentage band neutrophils (PBN), heart rate (HR), rectal temperature, respiratory rate (RR) above 20/min or not, pre-and post-operative cTnI-concentrations for 46 dogs with pyometra.

Case No	Weight (kg)	Age (years)	WBC (×109/ml)	Neutrophils (×109/ml)	PBN (%)	HR (bpm)	Temp (°C)	RR >20	Pre-op cTnI (μg/l)	Post-op cTnI (μg/l)	SIRS-pos
1	32	6.5	32	1.8	72	120	39.1	Y	13.2	6.4	+
2	7	11.5	7	-	-	128	40.4	Y	< 0.2	< 0.2	+
3	11	7	11.4	5.9	0	80	38.6	Y	< 0.2	< 0.2	-
4	28	11	28	6.9	89	104	39.6	-	0.7	0.7	+
5	11	8	11.3	10.9	12	-	39.9	-	1.2	0.4	+
6	33	10	33.5	9.7	0	110	38.8	Y	< 0.2	0.3	-
7	29	3.5	29	-	-	140	39.3	Y	< 0.2	< 0.2	+
8	30	8.5	30	15.5	43	144	39.9	N	2.0	1.0	+
9	12	13	12	26.8	91	-	38.3	N	0.3	0.4	+
10	10	12.5	9.7	1.6	12	134	39.9	N	< 0.2	< 0.2	+
11	27	7.5	26.6	-	-	128	39.4	N	< 0.2	0.4	+
12	35	7.5	35	14.2	0	140	39.2	Y	1.0	0.7	+
13	20	8	20.2	18.4	16	210	39.9	-	< 0.2	< 0.2	+
14	22	9.5	22.8	37.5	13	138	39.8	Y	0.3	< 0.2	+
15	9	12	9	23.6	94	100	40.9	Y	0.5	3.1	+
16	26	8	26.2	22.5	10	130	38.6	Y	< 0.2	0.3	+
17	22	9	22	9.8	53	-	40.7	Y	< 0.2	< 0.2	+
18	5	10	5.4	6.6	52	124	40.6	-	< 0.2	< 0.2	+
19	32	9	32.6	15	5	88	38.6	N	< 0.2	< 0.2	+
20	32	11	31.6	15.5	14	-	39	N	< 0.2	0.3	+
21	31	10	31	17.9	20	90	38.3	N	< 0.2	< 0.2	+
22	23	10	23	-	-	90	39.4	N	< 0.2	< 0.2	+
23	23	9	23.2	6.8	35	90	40.1	N	< 0.2	0.5	+
24	15	6	15.5	23.7	45	128	38.1	N	< 0.2	< 0.2	+
25	24	7	24.5	12.6	18	-	40.3	Y	< 0.2	< 0.2	+
26	24	8.5	23.8	9.5	20	140	39.2	Y	< 0.2	< 0.2	+
27	29	11	29	17.7	7	128	39.3	-	< 0.2	< 0.2	+
28	45	6	45	-	-	-	38.7	N	< 0.2	< 0.2	-
29	7	10	7.5	-	-	150	39.7	N	13.2	1.0	+
30	22	11	22.5	9.1	85	100	39.3	N	< 0.2	< 0.2	+
31	8	9	8.2	21.9	1	116	38.7	Y	< 0.2	< 0.2	+
32	17	8.5	17	11.1	74	124	40.3	N	< 0.2	0.3	+
33	20	4.5	20	9.6	0	112	39.6	Y	< 0.2	< 0.2	+
34	26	7.5	26.3	12.7	2	112	38.9	N	< 0.2	< 0.2	-
35	35	5	35.1	27	0	120	39.2	N	< 0.2	< 0.2	-
36	20	10	20	-	-	110	38.8	N	0.3	< 0.2	-
37	12	6	12	-	-	124	39.6	N	< 0.2	< 0.2	+
38	37	7	37.3	8.8	23	108	39.8	N	< 0.2	0.4	+
39	25	7	25	15.6	26	128	38.7	Y	0.4	0.5	+
40	33	8	33	8.8	0	90	38.5	Y	0.3	0.5	-
41	41	7	41	21	0	90	39.3	N	< 0.2	< 0.2	+
42	25	0.9	24.6	8	0	92	40.5	N	< 0.2	< 0.2	-
43	29	10	29.2	33.4	18	80	38.9	N	1.2	0.3	+
44	7	8	7.3	26.7	0	136	39.7	Y	< 0.2	< 0.2	+
45	17	7	17	5.1	0	140	39.9	N	< 0.2	< 0.2	+
46	15	4	14.7	13.5	3	120	38.9	N	< 0.2	< 0.2	-

The last column indicates if the dog was classified as SIRS-positive (+) or not (-) using the chosen SIRS criteria. Where values for HR and RR are missing they were not noted in the records. Values for Neutrophils and PBN are missing in 8 dogs.

### Postoperative cTnI concentrations

Two (13%) of the control dogs had increased cTnI concentrations the day after surgery (0.4 and 3 μg/l, respectively) (Table 2). Eighteen (39%) of the pyometra dogs had increased postoperative cTnI concentrations (range 0.3–6.4 μg/l) (Table 3). Of the 13 dogs with increased preoperative cTnI concentrations, 8 had lower, 4 had increased, and one had unchanged cTnI concentrations after surgery. In seven dogs with nondetectable cTnI concentrations at presentation, increased levels were demonstrated postsurgically.

Thus, in total, 20 dogs (43%) with pyometra had increased cTnI concentrations before or after surgery, and 7 dogs had pre- or postoperative cTnI concentrations of 1.0 μg/l or higher.

### Preoperative blood biochemistry

None of the 27 dogs that had serum ALAT concentration analysed had a concentration exceeding the upper reference range. Similarly, only one out of the 33 dogs that had serum creatinine concentration analysed had a concentration exceeding the upper reference range for creatinine. Finally, 6 of the 23 dogs that had serum ALP concentration and 6 out of the 20 dogs that had serum glucose concentration analysed had a concentration exceeding the upper reference range.

### SIRS groups and outcome

Eleven of the dogs with pyometra fulfilled all four SIRS criteria. Ten of the dogs fulfilled three criteria, 16 dogs fulfilled two criteria and 6 dogs one criterion for SIRS. Three dogs did not fulfil any of the SIRS criteria. Consequently the SIRS-positive group (two or more positive criteria) consisted of 37 dogs (80%) and the SIRS-negative group of 9 dogs (20%). All dogs except four were discharged within 48 hours of surgery. Reasons for delaying discharge in four dogs were resuturing of skin wound (n = 1), reduced general condition postoperatively (n = 1) and owner preference (n = 2). All dogs in the study recovered and survived the postoperative period (10 days).

### Comparison of cTnI concentrations and clinical parameters

**Figure 1:**
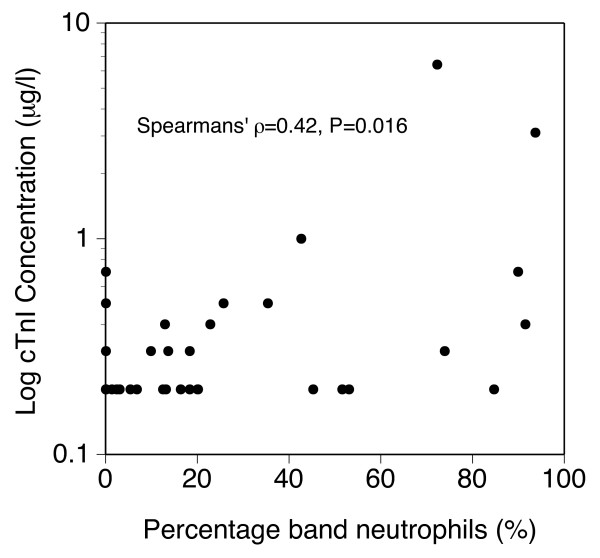
Scatterplot of postsurgical concentrations of cTnI by percentage band neutrophils in peripheral blood at presentation in 38 dogs (values missing in 8 dogs).

When the SIRS-positive and SIRS-negative groups were compared (both before and after surgery) there was no statistically significant difference in cTnI concentrations between the groups. The only significant association between the studied laboratory or clinical variables and cTnI concentration was preoperative percentage band neutrophils (PBN) and postoperative cTnI concentration (p = 0.016) (Figure 1). The preoperative PBN tended to be correlated with preoperative cTnI concentrations (p = 0.059).

## Discussion

In total, 20 out of 46 dogs (43%) with pyometra had increased concentrations of cTnI at some time during the study, which indicates that increased cTnI concentrations are common during the perioperative period in dogs with pyometra. This finding is supported by the results of our previous study in which increased preoperative cTnI concentrations were documented in 12% of 58 dogs with pyometra [18]. The present study is different from our previous one because, to our knowledge, this is the first study that documents both pre-and postoperative measurements of cTnI concentrations in dogs with pyometra. It should be pointed out that only 7 of the 20 dogs with increased concentrations of cTnI (10 out of the total 31 samples with increased concentrations) had values ≥ 1.0 μg/l and none of the dogs measured higher than 13.2 μg/ml, indicating that cTnI concentrations were mildly increased in the majority of cases. The lower limit of detection of cTnI in our assay is 0,2 μg/l. It is possible that the upper reference range for cTnI in dogs is lower than 0,2 μg/l and that a greater number of dogs would have had increased concentrations of cTnI if we had used a more sensitive assay, as recently described [19].

The clinical significance of mild increases in cTnI concentrations is currently unknown. Studies have shown an association between the degree of increase in cTnI concentrations and the size of myocardial infarctions in dogs [1,20]. However, it has been suggested that reduced renal function can cause an increase in cTnI in the absence of myocardial cell damage [21]. Most of the dogs in our study had preoperative creatinine concentrations determined. However, in 5 of the dogs with increased cTnI-concentrations the preoperative creatinine concentration was not known. Although alternative causes for mildly increased cTnI concentrations are possible [21], it is likely that the degree of increase of cTnI concentration in serum provides an estimate of the extent of myocardial damage in our dogs.

Detection of damaged myocardium may be useful for the clinician when managing a dog with pyometra because its presence could indicate that the dog might be at risk for adverse events such as ventricular arrythmias or unexpected death. Early identification of dogs at risk allows the clinician to take actions to avoid adverse cardiac events by monitoring the dog during the perioperative period and intervene early when indicated. Although none of the dogs had a history of known heart disease, one limitation of the study is that we did not rule out underlying subclinical cardiac disease in any of the participating dogs.

A possible cause for the increased cTnI concentrations could be the presence of endotoxins into the circulation. Elevated plasma endotoxin concentrations have been documented in female dogs with pyometra [22-24] and is thought to be responsible for some of the clinical signs [23]. In most cases of canine pyometra, *Escherichia coli (E. coli) *can be cultured from the uterus [22,25]. Like other Gram negative bacteria, *E. coli *can release endotoxin during growth or when they die [26]. Endotoxins bind to receptors on cell-membranes and induce inflammation and cytokine production [26]. Depending on the extent of endotoxin release, the result is varying manifestations of inflammation, from local to systemic, and cellular damage, which could potentially affect myocardial cells and thereby result in elevated serum concentrations of cTnI. Indeed, SIRS has been documented to be part of the clinical picture in 57% of 53 dogs [27] and 53% of 59 dogs [18] with pyometra. In our study, 37 out of 46 dogs (80%) fulfilled the chosen criteria for SIRS. However, we could not find an association between a diagnosis of SIRS and increased cTnI concentrations. These results are in accordance with our previous findings [18]. The diagnosis of SIRS is difficult because some of the clinical parameters used to determine its presence (body temperature, respiratory rate, heart rate, neutrophil count) are influenced by the excitement and stress caused by the visit to the animal hospital and by the disease as such. This influence would lead to a falsely high number of SIRS-positive dogs in the study population. The criteria for a positive diagnosis of SIRS used in this study were chosen to minimize the risk of failure to identify SIRS, thereby minimizing the risk of the serious consequences to the patient that can arise when this diagnosis is missed [6]. With a high sensitivity of 97% there is a concurrent low specificity (64%), explaining the risk of false positive diagnoses of SIRS in our population of dogs. It is possible that a correlation between cTnI concentrations and SIRS could be found if we could more reliably diagnose the presence of SIRS in an individual animal. C-reactive protein has been found to be a valuable marker of SIRS in dogs with pyometra and may be of value in future studies of dogs suspected to suffer from SIRS [28].

As a group, there was no significant change in the cTnI concentrations before and after surgery in the 46 dogs with pyometra. However, this lack of significance does not mean that changes have not occurred in individual dogs. Indeed, in 8 dogs the cTnI concentrations decreased after surgery and in 11 dogs the concentrations increased. A possible explanation for the decreased concentrations on the day after surgery could be the normal metabolism and elimination of cTnI from the body. The half-life of cTnI is reported to be 120 minutes [9]. One explanation for increased concentration of cTnI postsurgically could be that anaesthesia and surgery may cause further damage to the myocytes, in particular in individuals with systemic inflammation and impaired circulation, because of potential perioperative myocardial hypoxia. This phenomenon is well recognised in humans [10,29,30] but has, to our knowledge, not been shown to occur in dogs. Another possible reason for myocardial injury during anaesthesia could be direct toxic effects of the anaesthetic agents. Ongoing myocyte damage because of SIRS or inflammation induced by systemically released endotoxin could also contribute to elevated concentrations of cTnI postsurgically. Two of the healthy control dogs in our study (which all had undetectable concentrations of cTnI preoperatively) had increased concentrations of cTnI after surgery. This could possibly be explained by the aforementioned perioperative hypoxia (or toxicity) and subsequent myocardial cell damage.

The only studied variable that was significantly associated with cTnI concentrations was preoperative percentage of band neutrophils and postoperative cTnI concentrations (p = 0.016). The preoperative PBN and preoperative cTnI concentrations tended to be correlated (p = 0.059). A high PBN count in peripheral blood is considered a sign of a high demand of neutrophils in the tissues during inflammation [31]. The percentage of band neutrophils is, as mentioned earlier, one of the criteria used for the diagnosis of SIRS. Thus, the above-mentioned correlation might reflect myocyte damage caused by systemic inflammation.

## Conclusion

Mild to moderate increases in cTnI appears to be common in dogs with pyometra before and after surgery, but the clinical importance of this finding is uncertain. None of the studied clinical variables (including SIRS) were found to reliably predict increased preoperative cTnI concentrations. Because of the pre- and postoperative variation in cTnI concentrations it was not possible to identify a negative effect of anaesthesia and surgery on myocardial cell integrity. Consequently, analysing serum cTnI concentrations from dogs with pyometra could possibly help detect subclinical myocardial damage. Further studies are needed to investigate whether increased concentrations of cTnI are associated with a higher risk of perioperative complications.

## Competing interests

The authors declare that they have no competing interests.

## Authors' contributions

LP participated in the design of the study and carried out the practical recruitment of cases. She also drafted the manuscript. RH participated in the design of the study and the manuscript writing. JH participated in the design of the study and performed the statistical analysis. He also parcipitated in the writing of the manuscript. All authors read and approved the final manuscript.
